# Integrating Autism Care through a School-Based Intervention Model: A Pilot Study

**DOI:** 10.3390/jcm6100097

**Published:** 2017-10-19

**Authors:** Katherine Dang, Stephen Bent, Brittany Lawton, Tracy Warren, Felicia Widjaja, Michael G. McDonald, Michael Breard, Whitney O’Keefe, Robert L. Hendren

**Affiliations:** 1 Department of Epidemiology and Biostatistics, University of California—San Francisco, San Francisco, CA 94158, USA; kadang@alamedahealthsystem.org; 2 Department of Psychiatry, University of California—San Francisco, San Francisco, CA 94143, USA; stephen.bent@ucsf.edu (S.B.); bllawton@gmail.com (B.L.); tracy.warren@ucsf.edu (T.W.); felicia.widjaja@ucsf.edu (F.W.); 3 Oak Hill School, San Anselmo, CA 94960, USA; mmcdonald@myoakhill.org (M.G.M.); mbreard@myoakhill.org (M.B.); wokeefe@myoakhill.org (W.O.)

**Keywords:** integrated care, medical home, autism, online, Teacher Autism Progress Scale, school consultation

## Abstract

The purpose of this pilot study is to determine the feasibility of monitoring the progress of children with an autism spectrum disorder (ASD) both in school and at home to promote a school-based integrated care model between parents, teachers, and medical providers. This is a prospective cohort study. To monitor progress, outcome measures were administered via an online platform developed for caregivers and teachers of children (*n* = 30) attending a school specializing in neurodevelopmental disorders and using an integrated medical and education program. Longitudinal analysis showed improvements in a novel scale, the Teacher Autism Progress Scale (TAPS), which was designed to measure key autism-related gains in a school environment (2.1-point improvement, *p* = 0.004, ES = 0.324). The TAPS showed a strong and statistically significant correlation, with improvement in aberrant behavior (*r* = −0.50; *p* = 0.008) and social responsiveness (*r* = −0.70; *p* < 0.001). The results also showed non-statistically significant improvements in aberrant behavior, social responsiveness, and quality of life over time at both school and home. To assess feasibility of ongoing progress measurement, we assessed missing data, which showed caregivers were more likely to miss surveys during summer. Results demonstrate the value and feasibility of online, longitudinal data collection in school to assist with individualized education planning and collaborative care for children with ASD. Lessons learned in this pilot will support school outcomes researchers in developing more efficacious, collaborative treatment plans between clinicians, caregivers, and teachers.

## 1. Introduction

Autism spectrum disorder (ASD) is a complex neurodevelopmental disorder characterized by impairments in social interaction and restricted, repetitive patterns of behavior that is now estimated to affect one in 68 children [1]. Co-occurring developmental, psychiatric, and medical conditions are present in more than 70% of individuals with ASD, and include intellectual disability, language disorders, attention-deficit hyperactivity disorder (ADHD), anxiety, depression, obsessive-compulsive disorder, epilepsy, sleep disorders, and gastrointestinal problems [2,3,4]. Caring for children with ASD can be challenging and is estimated to cost $2.4 million over a lifespan [5]. Costs can include the direct costs of caring for an individual and the loss of work and productivity for caregivers. Children with ASD generally receive care from multiple providers in different domains, including behavioral therapy, speech and language therapy, physical therapy, occupational therapy, primary care, and medical specialties. The majority of children with ASD use three or more of these service providers and take at least one medication [6]. In addition, 88% of families report currently or recently treating their children with ASD with at least one complementary and alternative therapy [7].

Given the complexity of caring for a child with ASD, many experts have advocated for the use of a “medical home” or “family-centered” coordinated care model [8,9,10]. A medical home has been described as care that emphasizes “comprehensive services that are family centered, continuous, culturally sensitive, compassionate, and coordinated with other providers in the community” [11]. Prior studies have shown that children with special health care needs who receive care in the medical home have a number of improved outcomes including greater satisfaction, access to care, communication, fewer unmet needs, and reduced costs and negative impacts [12,13].

Unfortunately, most families of children with ASD do not receive coordinated care. The National Survey of Children found that 62% of children with ASD have unmet needs for care coordination [14]. In a study reporting the results of a focus group of parents of children with ASD, most parents felt that their physicians were too busy to provide coordinated care and assumed the role of care coordination themselves [8]. A separate study found that over half of families of children with ASD reported having a parent stop or reduce work because of their child’s needs, and over 25% spent greater than 10 h a week coordinating care [15].

There is clearly a largely unmet need to improve care coordination for children with ASD. Interestingly, the aforementioned studies that describe care coordination have limited or no description of coordinating care with schools or teachers. One study found that parents perceived little or no coordination between physicians and schools, and pediatricians reported never having been contacted by schools to discuss a child with ASD [8]. A recent review of studies examining academic performance of children with ASD found that these children often have specific areas of strength and weakness, suggesting that individualized assessment would allow for targeted educational programming [16]. The same review highlighted the lack of studies using measures that involved teachers and their observations within the classroom. This is a potentially missed opportunity for many reasons. Children who spend 7–10 h per day at school will have a variety of social interactions that may vary markedly from home and clinic settings. Also, their teachers can observe and monitor their changes over time. Therefore, care decisions made by parents or medical providers without input from the school environment may lack the rich information about symptom changes that take place at school. We therefore sought to expand the current care model—which separates care between caregivers, clinicians, and teachers—to a “three-component” model that emphasizes the need to have fluid communication between parents/caregivers, clinicians and teachers—the “three components” that typically comprise care for a child with ASD.

The underlying purpose of this pilot study was to investigate the feasibility of monitoring outcome measures of children with ASD using validated questionnaires that were administered to parents and teachers online. In collaboration with the teachers, we additionally developed two novel measures, the Teacher Autism Progress Scale (TAPS) and the Academic Progress Rating Scales (APRS), which we hoped to implement and test for initial utility at a school for children with ASD. Part of this effort involved developing a web-based, user-friendly platform that the participants are able to use to record regular changes in the child’s behavior, social responsiveness and progress in both the school and home environment. This process also involved the development of a novel outcome measure based on a collaborative process with teachers and designed to measure autism-specific changes that are observed in an educational environment.

Additionally, we sought to understand the factors affecting parent and teacher participation and attrition, an important part of assessing feasibility of our platform and program. Previous studies have indicated several potential factors that may be involved in study attrition, such as gender, race, and the number of children in the household [17]. To determine participation and response rates in our study and examine feasibility of an outcomes tracking database, such as the one assessed here, we analyzed several variables that may have contributed to missing data, including, but not limited to, these demographic factors.

## 2. Methods

### 2.1. Preparation and Collaboration

This study was carried out in accordance with the Declaration of Helsinki, and all of the procedures were approved by UCSF Committee on Human Research (#13–11086) prior to contacting any families, teachers, or students to participate in the study. Informed consent was obtained from all parents or guardians of subjects prior to their participation in the study. A partnership was formed with a local school specializing in the education of children with autism and other neurodevelopmental conditions affecting social interactions and behavior (Oak Hill School, San Anselmo, CA, USA, http://www.theoakhillschool.org/). The school is certified by the California Department of Education as a non-public, non-sectarian special education school (NPS), which allows it to contract with public school districts to provide educational and clinical services to students through their individualized education programs (IEPs). As of May 2017, more than 90% of students are publicly funded to receive services at the school.

### 2.2. Study Population

*Student population*: Oak Hill serves a heterogeneous population of children, adolescents, and young adults, all of whom have autism spectrum disorder (ASD) or other neurologically-based disorders of relating and communicating. A large number of co-morbidities are present in the population, which include seizure and tic disorders, intellectual disability, ADHD, obsessive-compulsive and anxiety disorders, learning disorders, cerebral palsy, language disorders, and other medical conditions (i.e., gastrointestinal disorders, fibromyalgia, leukodystrophy, etc.). The school has separate classrooms for children in grades K through 12 and class placements are affected by a number of factors, including verbal ability, age, and a careful behavioral and academic assessment.

### 2.3. Eligibility Criteria and Enrollment Process

Students were recruited through an e-mail announcement to the entire parent body of the school and through parent information sessions given at the school by the study investigators in 2013 and 2014. Researchers were careful to emphasize, both verbally and in writing, that study participation was not required and declining to participate would not affect the child’s educational program. All of the students with ASD enrolled in the school were considered as eligible. Our goal was to enroll as many children as possible in the pilot phase. ASD was defined as being present if the child had a diagnosis from a medical professional or if the student was determined by school staff and the study psychiatrist to meet *Diagnostic and Statistical Manual of Mental Disorders*, Fifth Edition (*DSM–5*) criteria for ASD [18]. School enrollment is currently 36 students ranging from six to 24 years of age. The only exclusion criterion was being unable or unwilling to complete study forms. Demographics of the enrolled study population, including measures of baseline severity, are described in the Results section and in Table 1.

### 2.4. Procedures

*School-based interventions:* Students receive special education instruction and customized on-site clinical programs which may include speech/language pathology, occupational therapy, and group and individual psychotherapy. A portion of students have received recent functional behavior analyses (FBAs), with school staff implementing positive behavior intervention programs. The school also offers arts-based therapies and adaptive arts instruction.

*Case conferences:* For each enrolled child, a case-conference session was scheduled by the school senior teacher coordinator with the study Principal Investigator, who is an expert in clinical evaluation and the care of children with ASD, and members of child’s team (special education teacher, psychotherapist, speech/language pathologist, occupational therapist, school administrator, research coordinator). These school consultation sessions included a review of the progress of the outcome measures in the web-based platform (parent and teacher) and verbal feedback from the teachers regarding the child’s behavior and social interactions. During each session, target behaviors/barriers/challenges were identified and the group discussed and developed an action plan to address these problem behaviors. Two case conferences were conducted each week except during holidays or other scheduling conflicts.

*Development of web-based platform:* A user friendly, web-based platform was developed for this study to allow for on-line data entry by parents and teachers and some management functions for the study research team. The development and implementation of this online platform is detailed in a previous study of children with ASD attending integrative medicine clinics [19]. The web-based platform was developed on 23 August 2013 and pilot tested during the 5-month period of September 2013 to January 2014. All of the outcome measures were loaded for system deployment on 16 May 2014, which was the beginning of formal recruitment and enrollment.

*Development and selection of outcome measures:* A literature review was conducted to identify the most appropriate outcome measures for the study. The outcomes were selected based on their validity, reliability, relevance, and sensitivity to the degree of change expected from any intervention for children with ASD [20]. The primary outcomes being measured were aberrant behavior, social responsiveness, and quality of life, which were assessed via the Aberrant Behavior Checklist (ABC), Social Responsiveness Scale (SRS), and Pediatric Quality of Life Scale (PEDS), respectively.

In addition, two internal scales, labeled the Teacher Autism Progress Scale (TAPS) and the Academic Progress Rating Scales (APRS), were developed by both the teaching staff and the research team. TAPS was developed to measure the weekly behavioral, social and functional progress in target areas of school-related child behavior and skills (Table S1 Supplementary), whereas APRS was designed to measure other key academic and functional domains on a quarterly basis. The content for both TAPS and APRS was generated in a series of focus groups combining UCSF research staff and Oak Hill School teaching staff, held in the fall of 2013. In this article, the weekly measure of TAPS will be analyzed and assessed for initial validity, whereas the quarterly APRS assessment will be implemented and analyzed in future studies.

Detailed past medical history for each child (including birth and family history, medical problems, prior medications, CIM, and other treatments) was also obtained via a parent intake questionnaire at baseline.

*Timeline:* Subjects were enrolled continuously between May 2014 and June 2016. Parents completed baseline measures upon enrollment, including the ABC, SRS, PEDS, and a medical history and demographic intake form. Parents and teachers completed the ABC and SRS at quarterly time points for the duration of the study. Parents additionally completed PEDS at these time points, and teachers additionally completed the APRS at these time points. Teachers also completed the TAPS on a weekly basis for all of the students enrolled in the study.

### 2.5. Statistical Analyses

The analyses determined *a priori* were to: (1) measure the change in outcome measures over time; (2) assess correlations between the TAPS and outcome measures, ABC and SRS scores; and (3) examine associations between baseline variables and missing data (termed “missingness”). All of the statistical analyses were performed using STATA 14 Statistical Software Package.

*Sensitivity analysis:* Multiple imputation and last-observation-carried forward were used as sensitivity analyses for missing data in all longitudinal analyses. Linear models were tested for linearity, normality of residuals, constant variance/heteroscedasticity and influential points using component-plus-residual (CPR) plots, qnorm and Kernel density plots, residual vs. fitted plots and DFBETAs, respectively. Influential points were tested using DFBETA and were excluded from the analysis if deemed to have high leverage and discrepancy.

*Longitudinal and correlation analyses*. Change in outcome measures over time was assessed via a mixed effects regression model and locally weighted scatterplot smoothing (LOWESS) curve. The magnitude and direction of association between TAPS and the outcome measures, aberrant behavior, and social responsiveness, was measured using Pearson’s correlation. Influential points following sensitivity analysis were excluded in the correlation analysis. The results for change of average total outcome scores per unit time by respondent type were adjusted for sex of the subjects, caregiver, teacher and grade of subjects.

*Missingness analysis.* To examine variables that might affect missing data, an odds ratio was derived to measure the association between the baseline covariates and the binary outcome of “missingness” (0 = not missing, 1 = missing). Several baseline variables (i.e., summer quarter, neurological disorders, and hospital visits) were included in a multivariate mixed effects model. Forward-stepwise selection was performed to select each additional variable based on a significance level of *p* < 0.2. For the baseline variables that had missing data, multiple imputation iterative chained equation (MICE) was used to replace the missing values [21].

*Analysis of respondent participation.* Form completion was calculated as the percentage of forms that were completed over the total number of forms assigned per quarter for caregivers/parents (total forms = 5) and teachers (total forms = 3). Response rate was calculated as the percentage of participants who answered any survey over the total number of participants enrolled at baseline for each time period a survey was filled out.

## 3. Results

### 3.1. Baseline Characteristics

During the two-year enrollment period from May 2014 to June 2016, 36 families were contacted by e-mail by the research coordinator and 30 children completed the consent form and enrolled in the study. Out of those who enrolled, 73.3% of the participants completed >50% of the questionnaire assessing baseline characteristics, which are outlined in Table 1. Out of the study population, 75% of the subjects were male, and average age at enrollment was 13.64 years old (SD = 2.92). Mean baseline scores on the ABC and SRS, used here as a baseline measure of ASD severity, were 68.2 (SD = 58.4, maximum possible score = 174) for the ABC and 162.3 (SD = 25.1, maximum possible score = 195) for the SRS, indicating a wide range of baseline social and behavioral abilities included in our study population. No subjects reported participation in any other studies for the duration of their participation in our study.

### 3.2. Case Conferences

A list of several recommendations developed by the school consultation team, composed of the school staff and the expert clinician, during the case conferences is displayed in Table 2. The different domains included recommendations for behavioral or classroom adjustments, forms of therapy (i.e., cognitive behavior therapy (CBT), psychoeducation, etc.) and biomedical interventions (i.e., selective serotonin reuptake inhibitors (SSRI), sulforaphane, anticonvulsant medication, etc.).

### 3.3. Exploratory Longitudinal Analysis

**Figure 1:**
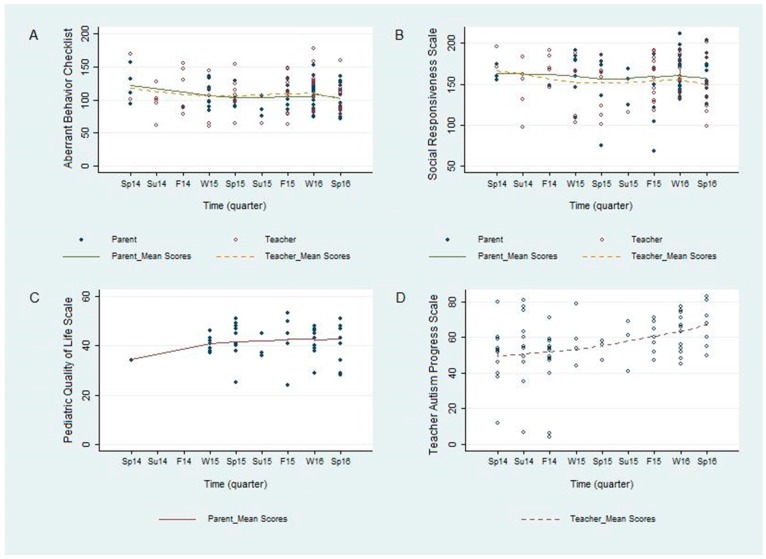
Longitudinal analysis of outcome scores by school quarter and respondent type: (**A**) change in aberrant behavior; (**B**) change in social responsiveness; (**C**) change in pediatric quality of life; and, (**D**) change in teacher autism progress scores (TAPS). Note: decrease in aberrant behavior (ABC) and social responsiveness (SRS) indicate improvement.

Results from the analysis of the change in outcome scores over time are displayed in Figure 1 and Table 3. We did not find statistically significant improvements in mean aberrant behavior scores (ABC), mean social responsiveness scores (SRS), or mean quality of life scores (PEDS) over time, though all three of these show a visual trend of improvement over time (Figure 1A–C). Mean ABC scores decreased (improved) non-significantly by −1.6 (95% CI −4.5 to 1.2; *p* = 0.26) and −0.7 (95% CI −4.7 to 3.3; *p* = 0.73) per school quarter based on caregiver and teacher ratings, respectively (Table 3). Mean SRS scores decreased (improved) non-significantly by −0.5 (95% CI −3.3 to 2.4; *p* = 0.74) per quarter based on parents’ responses (Table 3). In addition, pediatric quality of life (PEDS) improved non-significantly 0.5 (95% CI −0.3 to 1.3; *p* = 0.19) per quarter based on caregiver assessments (Table 3).

The weekly measure of total progress at school (TAPS), completed by teachers, increased (improved) over time (Figure 1D) Teacher ratings of total progress (TAPS) improved by 2.1 (95% CI 0.7 to 3.6; *p* = 0.004). This suggests that the TAPS, which was developed collaboratively by teachers and researchers, may be a potentially more sensitive tool to assess change in school-specific behaviors, whereas the other measures used in this study were not developed for specific use in a school environment.

### 3.4. Exploratory Correlation Analysis

**Figure 2:**
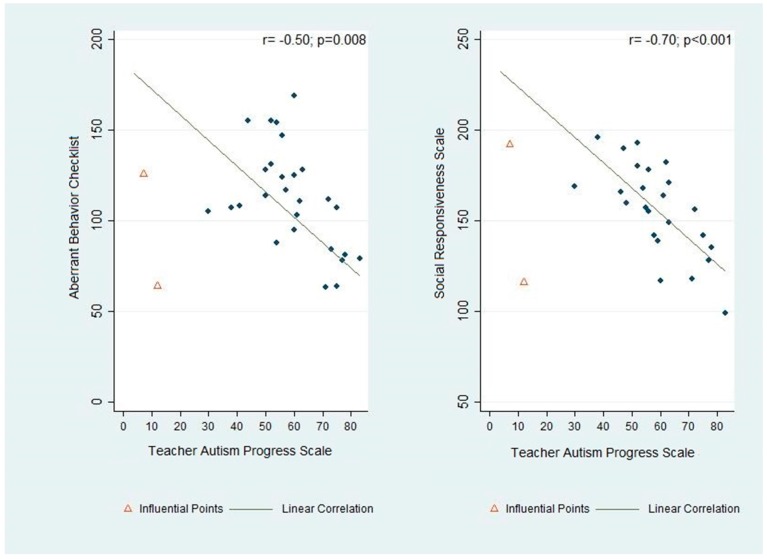
Analysis of the association between the progress of students at school and the outcome measures, aberrant behavior (left side) and social responsiveness (right side).

Figure 2 shows a statistically significant correlation between the TAPS and improvement in social responsiveness (SRS) (*r* = −0.70; *p* < 0.001), which is depicted as a decrease in SRS with increasing TAPS. Similarly, improvement (decrease) aberrant behavior scores was significantly correlated with improvement (increase) in TAPS scores (*r* = −0.50; *p* = 0.008).

### 3.5. Response Rate and Predictors of Study Missingness

Response rate, form completion, and results from the missingness analysis of targeted predictors are summarized in Supplementary Material (Table S2 Supplementary). The odds of respondents not filling out the SRS and ABC questionnaires were approximately seven times (OR = 6.9; *p* = 0.006) and eight times (OR = 8.7; *p* = 0.006) higher, respectively, in the summer quarters than when compared to in other quarters. The odds ratios for the other predictors did not reach statistical significance. The mean percentage of form completion was lowest in the summer quarters (<30%) for both teachers and caregivers. Response rates were observed to decrease from 93.3% to 30% for caregivers and 93.3% to 26.7% for teachers from baseline to the end of the first study period (Figure S1 Supplementary).

## 4. Discussion

The overarching goal of this study was to facilitate communication between caregivers, clinicians, and educators, in order to promote coordination of efficacious intervention programs for children with autism spectrum disorders. A coordinated care model such as ours has been advocated for in the literature [8,9,10], but we are unaware of any study assessing such a model in schools. In this study, we demonstrate the feasibility of implementing and tracking outcomes of this kind of model in a school. We were able to visually observe a trend of an overall improvement over time in validated outcome measures, including aberrant behavior, social responsiveness, and pediatric quality of life (Figure 1). We were also able to demonstrate a statistically significant improvement in the TAPS (Figure 1D), which was developed to measure the progress of the participants in a school setting in areas that are directly meaningful to the teachers, staff and clinicians at the school. These results were further supported by the correlational analysis, which showed a strong and statistically significant correlation between improvement on the TAPS and on the ABC and SRS (Figure 2). Prospectively, the observed results suggest that this novel internal instrument, TAPS, is a potentially reliable and sensitive measure for tracking the behavioral progress of children with autism at schools.

There were several limitations in our study that we hope to address with future research. Declining response rates are prevalent and unavoidable in longitudinal studies, and can increase the likelihood of nonresponse bias [17,22,23]. This pilot study showed that surveys assigned during the summer and perception of high survey burden (i.e., length, number and frequency of surveys) were the primary contributors to study dropout. Therefore, for future studies, the response rates could be improved with the allocation of additional resources to study retention. One likely solution would be to implement more effective community-engaged research strategies, such as including parents and teachers of children with ASD from outside the study population in the initial design of the data collection methods to help determine a feasible assessment schedule. Other methods will involve more consistent and frequent engagement (i.e., reminders, encouragement, help sessions, etc.) of the participants throughout the study.

An additional limitation of our study is the lack of standardization analysis of the TAPS. While we found statistically significant improvements in the TAPS over time and found statistically significant correlations between TAPS scores and ABC and SRS scores, we felt that a full validity and reliability analysis of the TAPS would be better done with higher response rates and a larger population than those that we experienced in this study. Because of this, we only intend the TAPS’s correlations with the ABC and SRS to be used as a preliminary indicator of its potential utility in ASD research. We hope to perform stronger analysis on this novel measure in future research.

Other limitations in our study include a small sample size, a wide age range in the subjects, a lack of exclusion criteria, and no control group, which can hopefully be controlled for in future studies. Due to the small population at the school, it was determined that the presence of exclusion criteria and a control group would result in a sample size too small to conduct the desired research. It is also possible that the results reported here might have been due to external factors outside of school interventions, a possibility that our methods do not address. Additionally, the significant improvement in the TAPS could have resulted from teachers’ potentially increased focus on TAPS-related domains during case conferences, causing them to focus more on brainstorming those specific TAPS-related challenges in their students and resulting in more improved scores on the TAPS than on the other measures assessed. We hope to expand upon this pilot in future studies with a large enough sample to be able to address these methodological issues.

Despite the limitations, results collected from this pilot study demonstrate proof-of-concept for systematic monitoring of the progress of children with autism in both the school and home environment. Children from this study population showed trends of improvement in aberrant behavior, social responsiveness, and pediatric quality of life over the study period, highlighting the value and utility of monitoring these outcomes as a way of measuring the effectiveness of school-based interventions. For example, it would be possible to examine changes in the entire school population or on an individual child level after the introduction of a new curriculum, a new program (e.g., gardening or music), a change of classrooms, or a change in medical therapy for a given child. This is the next step in our “medical home” outcomes research. Furthermore, we provide initial evidence that a novel outcome measure (TAPS) developed by teachers is sensitive to changes in child functioning in a school environment. The TAPS was strongly correlated with both the ABC and SRS and the only measure to show statistically significant changes over time, suggesting that it may be more sensitive for detecting meaningful behavioral and social change in a school environment than the ABC or SRS. We also identified areas that will likely improve retention and completion rates, including reduced participant burden and activities to promote community engagement. In conclusion, the data collected from this pilot study will improve collaborative care and potentially provide clinicians, caregivers, and educators with the tools and insight to collectively implement the most efficacious treatment plans for each individual child.

## Figures and Tables

**Table 1:** Baseline characteristics of study population.

Characteristic	% or Mean ± SD
**Subjects completing >50% of the baseline questionnaires**	73.3%
**Sex, *n* = 28**	
**Female, %**	25
**Male, %**	75
**Ethnicity, *n* = 11**	
**White, %**	73
**Other, %**	18
**Decline to state, %**	9
**Age, *n* = 22**	
**Mean (years) ± SD**	13.64 ± 2.92
**7–10 years old, %**	14% (*n* = 3)
**11–14 years old, %**	59% (*n* = 13)
**15–20 years old, %**	27% (*n* = 6)
**Medical co-disabilities, *n* = 22**	
**Yes, %**	59
**No, %**	27
**Don’t Know, %**	14
**Surgeries or hospital visits, *n* = 20**	
**Yes, %**	40
**Aberrant Behavior, *n* = 28 ^a^**	68.2 ± 58.4
**Social Responsiveness, *n* = 28 ^a^**	162.3 ± 25.1
**Pediatric Quality of Life, *n* = 25 ^b^**	42 ± 10.2
**Progress Report, *n* = 29 ^c^**	56.0 ± 15.2

**^a^** Includes both caregivers’ and teachers’ responses; **^b^** Based on caregivers’ responses only; **^c^** Based on teachers’ responses only.

**Table 2:** Examples of recommendations across different domains during the case conferences.

Participant	Target(s) Addressed	Behavioral/Classroom Recommendation	Therapy Recommendation	Biomedical Recommendation
**01**	Anxiety, communication, behavioral problems	Take breaks with sensory supports when needed	Augment language use with iPad, choice boards, and a feeling board	Start on NAC to reduce OCD behaviors; change Kepra
**02**	Anxiety	Reduce anxiety by restructuring school activities by difficulty level and time commitment	Start on CBT	Start on SSRI
**03**	Academic engagement, anxiety	Use affinities to access academic curriculum	Psychoeducation around subject’s comorbid OCD and anxiety	Start on sulforaphane
**04**	Behavioral problems, anxiety	One-on-one bonding time after school with a male teacher	Engage family in family therapy	Increase dose of SSRI
**05**	Anxiety, seizures	Supported volunteer placement at a local farm	Try journaling to help with anxiety	Change current anticonvulsant medication

SSRI = selective serotonin reuptake inhibitor; CBT = cognitive behavior therapy; OCD = obsessive compulsive disorder; NAC = N-acetyl cysteine.

**Table 3:** Change of average total outcome scores per unit time by respondent type.

Outcome Measure	Caregiver			Teacher		
	Mean Δ	95% CI	*p*	Mean Δ	95% CI	*p*
Aberrant Behavior Checklist	−1.6	−4.5 to 1.2	0.26	−0.7	−4.7 to 3.3	0.73
Social Responsiveness Scale	−0.5	−3.3 to 2.4	0.74	0.3	−1.8 to 2.3	0.80
Pediatric Quality of Life Scale	0.5	−0.3 to 1.3	0.19	-	-	-
Teacher Autism Progress Scale	-	-	-	2.1	0.7 to 3.6	0.004 *

* *p* < 0.05 indicates statistical significance; Note: adjusted for sex of subject, caregiver and teacher and grade of subject.

## Supplementary Materials

The following are available online at http://www.mdpi.com/2077-0383/6/10/97/s1, Figure S1: Overall study participation of caregivers and teachers were measured via (a) mean percentage of form completion per quarter; (b) percent response rate at each time period during the entire study. T: Teacher; C: Caregiver, Table S1: Teacher Autism Progress Scale (TAPS), Table S2: Exploratory analysis of the association between selected baseline variables and missingness.
