# Supplementary material for: Integrating Autism Care through a School-Based Intervention Model: A Pilot Study
Source: J Clin Med. 2017 Oct 19;6(10):97. doi: 10.3390/jcm6100097 (PMC5664012; doi:10.3390/jcm6100097)
Supplement: Supplementary file 1 [file jcm-06-00097-s001.zip › jcm-227410/Data and Analyses/Supplement - missingness analysis.docx]

| **STATA Output**   1. ABC Missingness Analysis 2. SRS Missingness Analysis |
| --- |
