# Supplementary figures and images for: Integrating Autism Care through a School-Based Intervention Model: A Pilot Study
Source: J Clin Med. 2017 Oct 19;6(10):97. doi: 10.3390/jcm6100097 (PMC5664012; doi:10.3390/jcm6100097)

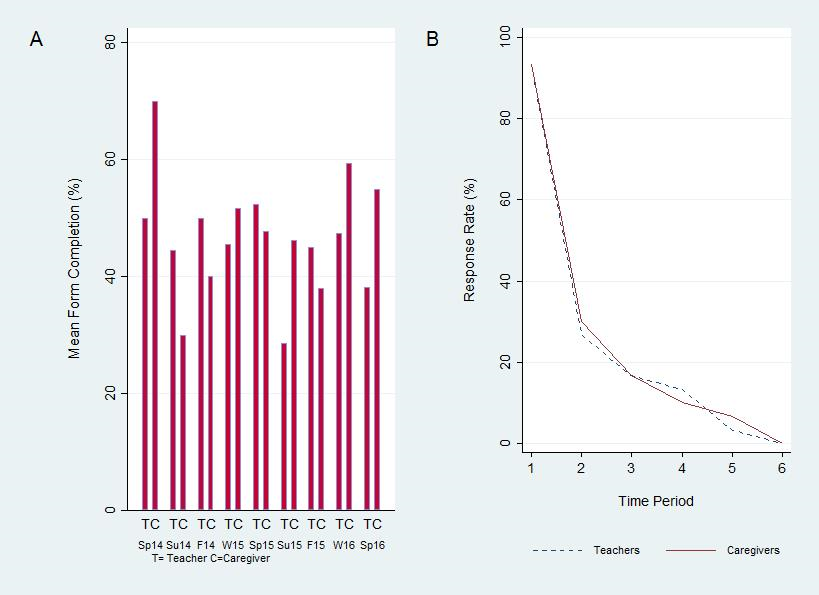

Supplement: Supplementary file 1 [file jcm-06-00097-s001.zip › jcm-227410/Supplementary tables and figures/(supplementary) Figure S1 - response rates over time.tif]
