# Supplementary material for: Integrating Autism Care through a School-Based Intervention Model: A Pilot Study
Source: J Clin Med. 2017 Oct 19;6(10):97. doi: 10.3390/jcm6100097 (PMC5664012; doi:10.3390/jcm6100097)
Supplement: Supplementary file 1 [file jcm-06-00097-s001.zip › jcm-227410/Supplementary tables and figures/(supplementary) Table S1 - Teacher Autism Progress Scale.doc]

**Table 4.** Teacher Autism Progress Scale (TAPS)

| **Domains** | **Sub-Domains** | **Questions** | **Items** |
| --- | --- | --- | --- |
| Language and Communication | Functional communication  Measures the frequency and quality of spontaneously initiated communication, verbal or non-verbal, with appropriate communicative intent). | 1. Over the past week, how often did [child] spontaneously initiate communications? | 1-Never  2-Sometimes  3-Occassionally  4-Often  5-Usually  6-Almost always  7-Always |
|  |  | 2. How would you rate the overall appropriateness of these communications? | 1-Absolutely inappropriate  2-Inappropriate  3-Slightly inappropriate  4-Neutral  5-Slightly appropriate  6-Appropriate  7-Absolutely appropriate |
|  | Social reciprocity  Measures the frequency and quality of verbal or nonverbal behaviors for social engagement and/or exchange. | 3. Over the past week, how often has [child] used verbal or nonverbal behaviors for the sole purpose of social engagement and/or exchange (NOT to meet own immediate needs, such as initiating social contact only when requesting)? | 1-Never  2-Sometimes  3-Occassionally  4-Often  5-Usually  6-Almost always  7-Always |
|  |  | 4. How would you rate the overall appropriateness of these social interchanges? | 1-Absolutely inappropriate  2-Inappropriate  3-Slightly inappropriate  4-Neutral  5-Slightly appropriate  6-Appropriate  7-Absolutely appropriate |
| Behavior | Aggressive behaviors  Measures the amount and frequency of aggressive behaviors. | 5. Over the past week, how often has [child] engaged in aggressive behaviors? | 1-Never  2-Sometimes  3-Occassionally  4-Often  5-Usually  6-Almost always  7-Always |
|  |  | 6. The student was aggressive in the following ways: (select all that apply) | Physically  Verbally  Towards peers  Towards staff  Towards community members  Self-injurious |
|  | Self-regulation  Measures [child]'s ability to regulate their emotions. | 7. Over the past week, how often was [child] able to regulate [his] emotions? | 1-Never  2-Sometimes  3-Occassionally  4-Often  5-Usually  6-Almost always  7-Always |
|  | Attention and focus  Measures the quality of [child]'s attention, ability to focus on the task at hand, and his/her ability to engage in tasks. | 8. Over the past week, how often was [child] able to attend and engage in tasks at hand? | 1-Never  2-Sometimes  3-Occassionally  4-Often  5-Usually  6-Almost always  7-Always |
|  | Determination/Grit  Measures the quality of [child]'s attention, ability to focus on the task at hand, and his/her ability to engage in tasks. | 9. Over the past week, during times that [child] was frustrated, how often was [he] able to persevere? | 1-Never  2-Sometimes  3-Occassionally  4-Often  5-Usually  6-Almost always  7-Always |
|  | Anxiety  Measures [child]'s level of anxiety. | 10. Over the past week, how would you rate [child]'s level of anxiety? | 1-None  2-Minimal  3-Mild  4-Moderate  5-Moderately severe  6-Severe  7-Very severe |
|  | Withdrawal  Measures [child]'s level of withdrawal. | 11. Over the past week, how would you rate [child]'s level of irritability and/or withdrawal? | 1-None  2-Minimal  3-Mild  4-Moderate  5-Moderately severe  6-Severe  7-Very severe |
|  | Overall Support | 12. Overall during the past week, [child] could participate in class activities with: | 1-No support  2-Minor support  3-Limited support  4-Moderate support  5-Extensive support  6-[Child] could not participate with class |
| Daily Living Skills/ Independence | Organization and Planning | 13. Over the past week, how organized was [child]'s materials and physical space? | 1-Never  2-Sometimes  3-Occassionally  4-Often  5-Usually  6-Almost always  7-Always |
|  | Caring for self  Measures [child]'s level of independence in the self-care domain, which includes hygiene, toileting, feeding. | 14. Over the past week, how often did [child] require help in the self-care domain? | 1-Never  2-Sometimes  3-Occassionally  4-Often  5-Usually  6-Almost always  7-Always |
|  | Community  Measures [child]'s level of independence in the community, including ability to cross the street independently, stay with the group, stand in line at the market, etc. | 15. Over the past week, how often did [child] require help in the community? | 1-Never  2-Sometimes  3-Occassionally  4-Often  5-Usually  6-Almost always  7-Always |
|  | Frequency help required in school activities  Measures [child]'s level of independence at school, across all school activities, including academic and non-academic tasks. | 16. Over the past week, how often did [child] require help in school activities? | 1-Never  2-Sometimes  3-Occassionally  4-Often  5-Usually  6-Almost always  7-Always |
