# Supplementary material for: Integrating Autism Care through a School-Based Intervention Model: A Pilot Study
Source: J Clin Med. 2017 Oct 19;6(10):97. doi: 10.3390/jcm6100097 (PMC5664012; doi:10.3390/jcm6100097)
Supplement: Supplementary file 1 [file jcm-06-00097-s001.zip › jcm-227410/Supplementary tables and figures/(supplementary) Table S2 - missing data analysis.doc]

**Table 5.** Exploratory analysis of the association between selected baseline variables and missingness.

|  | **Aberrant Behavior** | | | **Social Responsiveness** | | |
| --- | --- | --- | --- | --- | --- | --- |
| Covariate | OR | 95% CI | p | OR | 95% CI | p |
| Summer quarter ^a^ | 8.66 | 1.88 - 39.8 | .006* | 6.92 | 1.75 - 27.41 | .006* |
| Neurological issues ^a^ | 2.48 | .5 - 13.5 | .29 | 2.47 | .64 - 9.59 | .19 |
| Surgeries/hospital visits ^a^ | 1.29 | .4 - 4.7 | .70 | .98 | .31 - 3.10 | .97 |
| Other disabilities |  |  |  |  |  |  |
| No | ref | ref | ref | ref | ref | ref |
| Don't Know | .85 | .2 - 3.7 | .83 | .65 | .18 - 2.41 | .52 |
| Yes | .42 | .1 - 1.6 | .21 | .40 | .12 - 1.37 | .15 |
| Baseline parent intake form ^b^ |  |  |  |  |  |  |
| Not missing | ref | ref | ref | ref | ref | ref |
| Missing | 6.16 | .4 - 99.5 | .20 | 1.68 | .3 - 8.6 | .54 |
| Incomplete | .78 | .1 - 6.3 | .82 | .3 | .08 - 1.5 | .16 |
| Respondent type |  |  |  |  |  |  |
| Teacher | ref | ref | ref | ref | ref | ref |
| Parent/Caregiver | .20 | .05 - .7 | .02* | .47 | .19 - 1.18 | .11 |
| Grade of child |  |  |  |  |  |  |
| Pre-k to fifth | ref | ref | ref | ref | ref | ref |
| 6th-8th | .32 | .07 - 1.4 | .13 | .34 | .08 - 1.48 | .34 |
| 9th-12th | .37 | .06 - 2.2 | .27 | .40 | .07 - 2.23 | .30 |

**^a^** Reference is "No."

**^b^** Reference is "Female."

Note: adjusted for sex of respondents and subjects.
