# Supplementary material for: Integrating Autism Care through a School-Based Intervention Model: A Pilot Study
Source: J Clin Med. 2017 Oct 19;6(10):97. doi: 10.3390/jcm6100097 (PMC5664012; doi:10.3390/jcm6100097)
Supplement: Supplementary file 1 [file jcm-06-00097-s001.zip › jcm-227410/Supplementary tables and figures/Supplementary Figure S1 caption.doc]

**Supplementary Figure 3 Caption:**

**Figure 3**. Overall study participation of caregivers and teachers were measured via (a) mean percentage of form completion per quarter; (b) percent response rate at each time period during the entire study. T: Teacher; C: Caregiver
